# Diagnostic yield of FAP-guided positron emission tomography in thyroid cancer: a systematic review

**DOI:** 10.3389/fmed.2024.1381863

**Published:** 2024-03-25

**Authors:** Alessio Rizzo, Domenico Albano, Francesco Dondi, Martina Cioffi, Barbara Muoio, Salvatore Annunziata, Manuela Racca, Francesco Bertagna, Arnoldo Piccardo, Giorgio Treglia

**Affiliations:** ^1^Department of Nuclear Medicine, Candiolo Cancer Institute, FPO – IRCCS, Turin, Italy; ^2^Division of Nuclear Medicine, Università degli Studi di Brescia and ASST Spedali Civili di Brescia, Brescia, Italy; ^3^Nuclear Medicine Unit, Department of Medical Sciences, AOU Città della Salute e della Scienza, University of Turin, Turin, Italy; ^4^Clinic of Medical Oncology, Oncology Institute of Southern Switzerland, Ente Ospedaliero Cantonale, Bellinzona, Switzerland; ^5^Unità di Medicina Nucleare, GSTeP Radiopharmacy - TracerGLab, Dipartimento di Diagnostica per Immagini, Radioterapia Oncologica ed Ematologia, Fondazione Policlinico Universitario A. Gemelli, IRCCS, Rome, Italy; ^6^Department of Nuclear Medicine, E.O. “Ospedali Galliera,” Genoa, Italy; ^7^Clinic of Nuclear Medicine, Imaging Institute of Southern Switzerland, Ente Ospedaliero Cantonale, Bellinzona, Switzerland; ^8^Faculty of Biology and Medicine, University of Lausanne, Lausanne, Switzerland; ^9^Faculty of Biomedical Sciences, Università della Svizzera Italiana, Lugano, Switzerland

**Keywords:** thyroid cancer, FAPI, PET, nuclear medicine, oncology, systematic review

## Abstract

**Background:**

Several recent studies have proposed the possible application of positron emission tomography/computed tomography (PET/CT) administering radiolabelled fibroblast-activation protein (FAP) inhibitors for various forms of thyroid cancer (TC), including differentiated TC (DTC), and medullary TC (MTC).

**Methods:**

The authors conducted an extensive literature search of original studies examining the effectiveness of FAP-guided PET/CT in patients with TC. The papers included were original publications exploring the use of FAP-targeted molecular imaging in restaging metastatic DTC and MTC patients.

**Results:**

A total of 6 studies concerning the diagnostic yield of FAP-targeted PET/CT in TC (274 patients, of which 247 DTC and 27 MTC) were included in this systematic review. The included articles reported high values of FAP-targeted PET/CT detection rates in TC, ranging from 81 to 100% in different anatomical sites and overall superior to the comparative imaging method.

**Conclusion:**

Although there are promising results, the existing literature on the diagnostic accuracy of FAP-guided PET in this context is still quite limited. To thoroughly evaluate its potential significance in TC patients, it is needed to conduct prospective randomized multicentric trials.

## 1 Introduction

Differentiated thyroid cancer (DTC) is the leading malignant tumour affecting the endocrine system, and its global occurrence continues to increase annually due to the implementation of enhanced screening methods, such as neck ultrasonography and fine needle aspiration biopsy in everyday clinical practice, for detecting and characterising small thyroid nodules (1). Most of these TCs encompass small and asymptomatic papillary TCs (PTCs) belonging to a significant subclinical group of slow-growing tumours (2) and high-risk DTCs in a lower percentage (3). Adjuvant post-surgical Radioiodine (RAI) therapy and relative post-therapeutic whole-body scan performed after RAI administration have traditionally been crucial in assessing the extent of tumour burden in high-risk DTC and the ability of residual or recurrent illness to concentrate RAI (3). Regrettably, only around two-thirds of patients with metastatic DTC exhibit uptake of RAI in their lesions. Conversely, the remainder of patients either develop metastases that do not exhibit significant RAI uptake on post-therapeutic whole-body scan (or lose the ability to concentrate it) or experience disease progression after RAI treatment (3, 4). As a result of this statement, the concept of RAI-refractoriness (RAI-R) was introduced in the literature. According to the latest American and European Thyroid and Nuclear Medicine Societies, RAI-R is defined as follows: patients with abnormal thyroglobulin (Tg) levels or evidence of disease in other diagnostic examinations without RAI concentration on a diagnostic or post-therapeutic RI scan; tumour foci that show RAI uptake while others do not; progressive disease despite evidence of RAI uptake in DTC lesions (3).

Concerning the diagnostic instrumental investigations currently employed to manage RAI-R DTC patients, whole-body contrast-enhanced computed tomography (CT) is the most diffused examination due to its ability to assess the development of locally recurrent invasive disease, lymph node and distant metastases as well as its worldwide availability and cost-effectiveness (5). However, several authors have observed how fluorine-18 fluorodeoxyglucose ([^18^F]FDG) positron emission tomography (PET)/CT may play a role in the management of these patients, particularly as a prognosis predictor, despite its variable accuracy, which is usually affected by different pathology features such as tumour dedifferentiation and burden (3, 6, 7).

Among the variety of tumours which can onset in the context of thyroid gland, it does worth mention the medullary thyroid cancer (MTC), which is a rare neuroendocrine neoplasm originating from the para-follicular C-cells of the thyroid and encompasses for 3 to 10% of all TCs (8–10). Calcitonin is the most commonly used serum marker for screening and monitoring patients diagnosed with MTC (11). When MTC is detected, the metastatic spread to the cervical lymph nodes is a frequent condition (12). Regarding MTC, CT is the primary cross-sectional imaging technique used to evaluate the severity of the disease in individuals with elevated calcitonin levels, particularly for lymph nodes and liver metastases (although magnetic resonance imaging is more effective in detecting bone lesions) (13, 14). Regarding the molecular imaging of MTC, PET/CT is a highly beneficial nuclear medicine technology in comparison to conventional scintigraphic techniques. The PET radiopharmaceuticals most typically utilized in MTC restaging (when blood tumour marker levels rise) are [^18^F] dihydroxyphenylalanine ([^18^F]DOPA), [^18^F]FDG and [^68^Ga]Ga-labelled somatostatin analogs and, among them, [^18^F]DOPA has the best detection rate (15, 16).

Overall, there is an urgent need of more accurate molecular imaging methods focused on new targets for evaluating RAI-R DTC and MTC.

For several decades, carcinomas were thought to be made up of altered cells with cell-autonomous hyper-proliferative and invasive survival features. Nevertheless, the tumour microenvironment (TME), which includes tumour-associated stromal cells and the extracellular matrix, is also crucial in tumour invasion and metastases onset, promoting cell migration (17–19); for example, cancer-associated fibroblasts (CAFs) are a significant component of the tumour stroma. CAFs, also known as reactive fibroblasts or myofibroblasts, are found in a variety of malignant tumours, including head and neck malignancies other than TC (20, 21). FAP expression may also variate according to the solid tumours’ grading, such as in prostate cancer (22, 23). Despite CAFs being stromal cells, which are part and parcel of neoplastic lesions, they do not express epithelial, endothelial, or leukocyte markers and are notably devoid of oncogene mutations (24). Finally, CAFs have been found to express a variety of receptors on their cell membrane, including alpha-smooth muscle actin and fibroblast-activating protein (FAP) (25). FAP expression is typically slight in physiologic adult tissues but significantly higher in sites undergoing tissue remodelling, such as malignancies (26). According to these findings, FAP became an attractive target for molecular imaging of various cancers and non-oncological disorders. Subsequently, different radiolabelled FAP inhibitors (FAPi) have been synthesised in order to assess the *in vivo* expression of FAP by PET imaging in various malignancies, including TC (27). The purpose of this systematic is to thoroughly evaluate the diagnostic performance of FAP-guided PET imaging in detecting TC lesions in different clinical scenarios.

## 2 Materials and methods

### 2.1 Protocol and review question

A preconceived protocol guided the development of the current systematic review (28). Namely, it was based on the “Preferred Reporting Items for a Systematic Review and Meta-Analysis” (PRISMA 2020 statement) for its put in writing (29). Supplementary Table 1 reports the thorough PRISMA checklist. The present systematic review was not registered in any comprehensive listing of systematic review protocols (e.g., PROSPERO).

The first step was defining a review question according to the PICO (Population, Intervention, Comparator, Outcomes) framework: which is the diagnostic yield (outcome) of FAP-guided PET imaging (intervention) in patients with TC (patient) compared with other imaging methods (comparator)? This predefined review question has guided the choice of eligibility criteria for the inclusion of pertinent studies in the systematic review.

The comprehensive literature search, study selection, quality assessment and data extraction were all performed by two reviewers (AR and GT) independently. Any disagreements among the reviewers were resolved through a discussion with a third reviewer.

### 2.2 Literature search strategy and information sources

As stated, the authors searched for articles concerning the employment of FAP guided PET in the management of TC using two distinct electronic bibliographic databases (Cochrane Library and PubMed/MEDLINE).

Taking into account the predefined review question, a search algorithm was created based on a combination of the following terms: (A) “FAP” OR “FAPi” AND (B) “thyroid.” Terms such as “PET,” “positron,” and “cancer” were deliberately excluded from the search algorithm since the authors agreed on opting for a more sensitive research string than for a specific one, trying to collect all the articles concerning the preconceived topic.

No restrictions were applied regarding the articles’ language or publication year. Moreover, reviewers screened included studies’ references, searching for additional eligible articles considering the review question. The literature search was last updated on 06 December 2023.

### 2.3 Eligibility criteria

This systematic review deemed clinical trials reporting data on the diagnostic yield of FAP-guided PET imaging in TC patients appropriate for inclusion. Editorials, letters, reviews, comments, case reports, minor case series, and original investigations on different topics than the preconceived one (including pre-clinical studies) were excluded. Moreover, since the present review had the aim to assess the potential role of FAP-guided PET in TC diagnostics, studies concerning the role of FAP-targeting radiopharmaceuticals as radioligand therapy agents were not considered as papers in the field of interest.

### 2.4 Selection process

At least two review authors (AR and GT) separately examined the titles and abstracts from the list of records generated using the search string in the selected bibliographic databases. They chose the studies eligible for the systematic review based on the stated inclusion and exclusion criteria, explaining their reasoning for each selection.

### 2.5 Data collection process and data extraction

The reviewers (AR and GT) gathered data from all of the included studies, taking advantage of full-text, tables, and figures regarding general study information (authors, publication year, country, study design, funding sources); patients’ characteristics (sample size, age, sex ratio, clinical setting, histological TC subtypes, serum markers levels); and index text characteristics (employed radiopharmaceuticals, hybrid imaging protocol, administered radiopharmaceutical activity, uptake time).

### 2.6 Quality assessment (risk of bias assessment)

QUADAS-2, a methodology for evaluating quality in diagnostic test accuracy studies, was chosen to analyse the risk of bias in individual studies and their applicability to the review question (28). Two authors (AR and GT) graded the research in the systematic review based on the potential for bias in four areas (patient selection, index test, reference standard, and flow and timing) and applicability in three categories (patient selection, index test, and reference standard).

## 3 Results

### 3.1 Literature search and study selection

**FIGURE 1:**
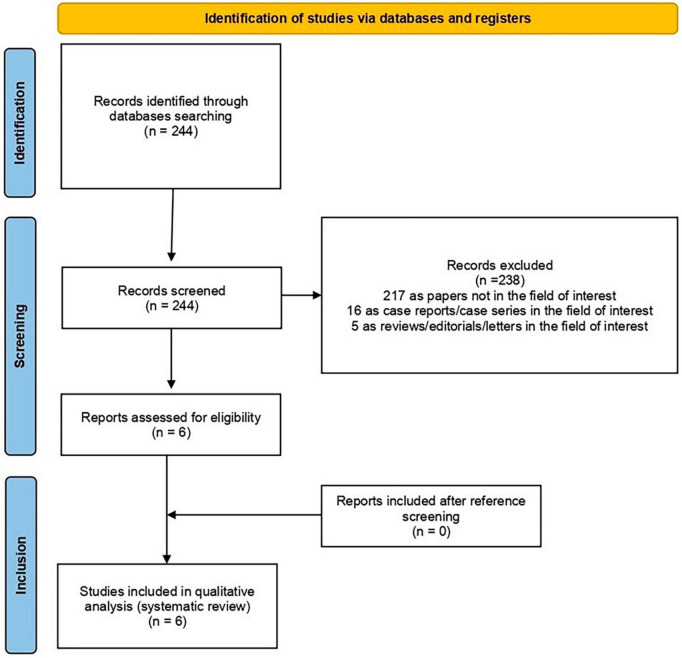
Comprehensive overview of the study selection process for the systematic review.

The literature search was updated on 06 December 2023 and yielded 244 records. Subsequently, 238 publications were excluded based on the previously stated selection criteria. Of these, 217 were deemed irrelevant to the field of interest, five were reviews, editorials, book chapters, or letters related to the analysed topic, and 16 were case reports within the field of interest. At the end, six publications were judged as eligible by the review authors and they were included in the qualitative synthesis based on the preconceived inclusion criteria (30–35). Reviewers were unable to locate any further appropriate publications by examining the references of these articles. Figure 1 provides a concise overview of the approach used to select the studies for this analysis.

### 3.2 Study characteristics

The present systematic review includes six studies satisfying the inclusion criteria. These studies involve 247 TC patients and are comprehensively analysed in Tables 1–3 (30–35). The chosen studies were published by Chinese (3/6), Indian (2/6), and Turkish (1/6) groups during the years 2022 and 2023. Half of the studies reported in the analysis utilized a prospective design, while the remaining half conducted a retrospective analysis of their case studies. Each trial included in the review was performed in a single Institution. Additionally, two studies mentioned the sources of funding in their text.

**TABLE 1:** General study information.

References	Country	Study design/number of involved centres	Funding sources
Chen et al. (30)	China	Prospective/single centre	National Natural Science Foundation of China; Natural Science Foundation of Fujian; Fujian Provincial Health Commission Science and Technology and Programme.
Fu et al. (31)	China	Prospective/single centre	None declared
Mu et al. (32)	China	Prospective/single centre	Guangxi Health Commission; Key Laboratory of Nanomedical Technology (Education Department of Fujian Province); School of Pharmacy, Nano Medical Technology Research Institute, Fujian Medical University; Key Laboratory for Endocrine-Related Cancer Precision Medicine of Xiamen.
Sayiner et al. (33)	Türkiye	Retrospective/Single centre	None declared
Ballal et al. (34)	India	Retrospective/single centre	None declared
Ballal et al. (35)	India	Retrospective/Single centre	None declared

**TABLE 2:** Patient key characteristics and clinical settings.

References	Sample size	Mean/median age (years)	Gender (male %)	Clinical setting (no. patients)	Histopathological TC subtypes (no. patients)	Mean/Median Tg / calcitonin	Comparative imaging
Chen et al. (30)	24	Mean: 49.6 ± 10.5	29%	RAI-R DTC	22 PTC 2 n.a.	Mean Tg: 791.7 ng/mL	CT
Fu et al. (31)	35	Median: 44	51%	Restaging DTC	32 PTC 2 FTC 1 Hürthle TC	Median Tg: 60.2 ng/mL	[^18^F] FDG PET/CT
Mu et al. (32)*	42	Median: 45	38%	Relapsing DTC	36 PTC 5 FTC 1 Hürthle TC	Tg < 10 ng/mL in 24 patients Tg > 10 ng/mL in 18 patients	TxWBS, [^18^F] FDG PET/CT
Sayiner et al. (33)	29	Mean: 45.8 ± 16.4	24%	Relapsing DTC	25 PTC 4 PDTC	Mean Tg: 1552.9	[^18^F] FDG PET/CT
Ballal et al. (34)	117	Mean: 53.2 ± 11.7	42%	RAIR-R DTC	69 PTC 17 FTC PDTC	Median Tg: 183 ng/mL	[^18^F] FDG PET/CT
Ballal et al. (35)	27	Mean: 42.4 ± 13.2	78%	Staging and restating MTC	27 MTC	Median calcitonin: 666.5 pg/mL	[^68^Ga]Ga-DOTANOC

CT, computed tomography; DTC, differentiated thyroid cancer; FDG, fluorodeoxyglucose; FTC, follicular thyroid cancer; MTC, medullary thyroid cancer; n.a., not available; PET, positron emission tomography; PDTC, poorly differentiated thyroid cancer; PTC, papillary thyroid cancer; RAI-R, radioiodine refractory; TC, thyroid cancer; Tg, thyroglobulin; TxWBS, post-therapeutic radioiodine whole-body scan.

*Only a part of the included patients underwent comparative imaging (13 patients and 11 patients were submitted to TxWBS and [^18^F] FDG PET/CT).

**TABLE 3:** Index test key characteristics.

References	Tracer	Hybrid imaging	Tomograph	Administered activity	Uptake time (minutes)	Image analysis
Chen et al. (30)	[^68^Ga]Ga-DOTA-FAPi-04	Analogic PET/CT	Biograph mCT 64 (Siemens^®^)	1.85–2.22 MBq/Kg	30	Qualitative, semiquantitative (SUV_max_, SUV_mean_, TBR)
Fu et al. (31)	[^68^Ga]Ga-DOTA-FAPi-04	Digital PET/CT	Discovery MI (GE^®^)	1.8–2.2 MBq/Kg	60	Qualitative, semiquantitative (SUV_max_)
Mu et al. (32)	[^18^F]FAPi-42	AnalogicPET/CT	Ingenuity TF (Philips^®^)	215 MBq	60	Qualitative, semiquantitative (SUV_max_, TBR)
Sayiner et al. (33)	[^68^Ga]Ga-DOTA-FAPi-04	Digital PET/CT	Discovery IQ (GE^®^)	185–222 MBq	30	Qualitative, semiquantitative (SUV_max_)
Ballal et al. (34)	[^68^Ga]Ga-DOTA.SA.FAPi	Analogic PET/CT	Discovery 710 (GE^®^)	180 MBq	60	Qualitative, semiquantitative (SUV_max_, SUL_peak_)
Ballal et al. (35)	[^68^Ga]Ga-DOTA.SA.FAPi	Analogic PET/CT	Discovery 710 (GE^®^)	185 MBq	60	Qualitative, semiquantitative (SUV_max_, SUL_peak_)

CT, computed tomography; FAPi, fibroblast activation protein inhibitor; MBq, MegaBecquerel; PET, positron emission tomography; SUL, standard uptake value corrected for lean body mass; SUV, standardized uptake value; TBR, target-to-background ratio.

Table 2 provides details regarding the TC patients included across the different studies. The number of individuals involved ranged from 24 to 117, with an average age of 42.4 to 53.2 years. The proportion of male participants varied from 24 to 51%. The index test was only employed for restaging RAI-R DTC patients in two papers (29, 32). In three research, it was utilized for restaging DTC patients regardless of their refractoriness to RAI (31–33). The last study focused on employing the index test for both staging and restaging MTC patients (35). Regarding histologic subtypes, five studies included individuals with DTC, with the most common variant being papillary thyroid cancer (PTC) with 184 patients (30–34). The remaining research specifically targeted patients who had been diagnosed with MTC (33). Regarding the five investigations dealing with DTC, the average value of Tg varied from 60 to 1552 ng/mL (30–34). Conversely, the only study including patients diagnosed with MTC revealed a central value of calcitonin at 666.5 pg/mL (35). Ultimately, tree research studies compared the results obtained from the index test using only [^18^F]FDG PET/CT (31, 33, 34); another publication compared the index test to [^18^F]FDG PET/CT and post-therapeutic RAI whole-body scan (32); one paper utilised contrast-enhanced CT as a comparator (30), while the last paper employed [^68^Ga]Ga-DOTANOC PET/CT (35).

The index test features exhibited substantial variation among the studies taken into account, as seen in Table 3 of the present review. Three experiments utilised [^68^Ga]Ga-DOTA-FAPi-04 (30, 31, 33), two studies employed [^68^Ga]Ga-DOTA-SA-FAPi (34, 35), and one utilised [^18^F]FAPi-42 (32). The administered activity varied between 180 and 222 MBq when measured using absolute values and between 1.8 and 2.22 MBq/Kg when measured using relative values. Furthermore, there was a time interval of 30 to 60 min between the administration of the experimented radiopharmaceutical and the PET imaging procedure. All the experiments utilised PET/CT as a hybrid imaging technique. PET scans in all the included studies were analysed using qualitative and semiquantitative techniques (30–35). The PET metrics assessed in the included publications were the target-to-background uptake ratio (TBR), as well as the maximal and mean standardised uptake values (SUVmax and SUVmean) of the pathological findings under examination. Regarding the PET metrics documented in the research, two articles authored by the same group presented the standardised uptake value adjusted for the lean body mass of the analysed lesions (SULpeak) (34, 35).

### 3.3 Risk of bias and applicability

**FIGURE 2:**
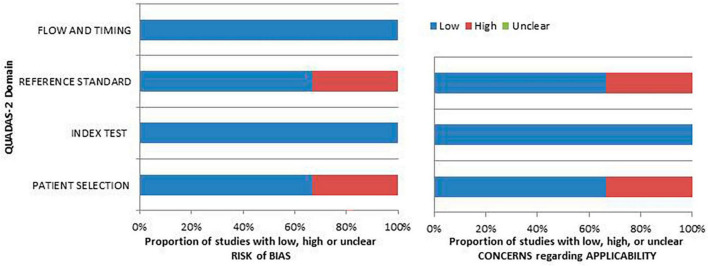
An iconographic summary of the quality assessment carried out with the QUADAS-2 tool. The researchers classified the papers included in the qualitative synthesis according to their degree of bias or applicability concerns for specific areas indicated on the ordinate axis. By contrast, the abscissa axis displays the percentage of studies. Based on the graph, about the 40% of the assessed studies demonstrate a notable risk of bias in the “patient selection” and “reference standard” domains.

The authors assessed the risk of bias and the relevance of the included publications using the QUADAS-2 tool, extracting the information presented in each investigation. Figure 2 presents the findings concerning the quality evaluation as well as the concerns regarding the applicability of the included research.

### 3.4 Results of individual studies (qualitative synthesis)

None of the reports included in the analysis revealed any negative effects following the injection of FAP-targeting radiopharmaceuticals (30). None of the included papers evaluated the inter-reader agreement of FAP-targeted PET images in assessing thyroid malignancies. All the studies dealing with DTC evaluated the uptake of FAP-targeting radiopharmaceuticals in both regional and metastatic sites: in the majority of reports, the tumour uptake was reported to be higher than the surrounding background. Concerning the semiquantitative metrics, average SUVmax reported values ranged from 4.2 and 12.6 for local recurrences and varied between 4.1 and 9.1 for metastatic lesions, including neck lymph nodes and distant metastases in lung, bone, liver and pleura. The high heterogeneity observed among the included studies may be explained by differences in the administered FAP-targeting radiopharmaceutical forms, radiolabelled with different positron-emitters radionuclides, in the PET devices employed and in the uptake times (30–35).

Among the studies exploring the role of FAP-targeted PET in DTC patients, one expressed the uptake of the lesions using standardized uptake value corrected for lean body mass (SULpeak) as the unit of measurement; subsequently, it was not feasible to compare its results to the other included studies. Among the included papers, four assessed the FAP-guided PET diagnostic yield in the detection of local recurrences as well as of distant metastases in DTC based on a per-patient analysis: two of them reported an overall value of detection rate (DR) of approximately 85%, one reported an overall sensitivity and specificity of 96 and 50%, respectively, and one assessed a DR for lymph node metastases of 86%, for lung lesions of 81.7% and bone secondaries of 100%. Conversely, only two of the included studies performed a per lesion analysis, with one paper reporting a sensitivity and specificity for neck lesions of 83 and 42%, respectively, and a DR for distant metastases of 79%, and the other reporting a DR for lymph node metastases of 95.4% (30–34).

When compared to [^18^F]FDG PET, the index test overall showed a superior diagnostic performance; FAP-guided PET was indeed able to reveal a higher number of lesions both in lymph nodes as well as in lung, bone, and liver; however, its superiority was not statistically relevant in most of the cases. The results of the included papers dealing with DTC, including semiquantitative metrics, sites of the lesions, and diagnostic yield of the index test, are reported in Table 4 (30–34).

**TABLE 4:** Main results of the included studies.

References	Lesions’ PET metrics	Lesions site	Diagnostic performance		Outcome
			Per patient	Per lesion	
Chen et al. (30)	Mean SUV_max_ Lymph node: 7.06 ± 0.39 Lung: 6.39 ± 0.91 Bone: 4.01 ± 0.48	Lymph node Lung Bone Pleura	DR: 87.5%	n.a.	DTC lesions showed intermediate-to-high uptake on FAP-guided PET images.
Fu et al. (31)	Median SUV_max_ Local recurrence: 12.6 Lymph node: ranging from 6.0 to 9.1 depending on the region Lung: 1.7 Bone: 6.0	Local recurrence Lymph node Lung Bone Liver	Sensitivity: 96% Specificity: 50%	Neck lesions sensitivity: 83% specificity: 42% Distant metastases DR: 79%	FAP-guided PET has superior diagnostic performance over FDG PET in a per-lesion analysis.
Mu et al. (32)	Median SUV_max_ Local recurrence: 4.2 Lymph node: 3.9 Lung: 1.3	Local recurrence Lymph node Lung Bone	n.a.	n.a.	FAP-guided PET had comparable performance to FDG PET.
Sayiner et al. (33)	Mean SUV_max_ All lesions: 7.5 ± 3.41	n.a.	DR: 86.2%	n.a.	FAP-guided PET was able to individuate more positive patients than FDG PET.
Ballal et al. (34)	Median SUL_peak_ Lymph node: 6.86 Lung: 5.64 Bone: 8.24	Primary tumour Local recurrence Lymph node Lung Pleura Bone Liver Brain	Lymph node DR: 86% Lung DR: 81.7% Bone DR: 100%	Lymph node DR: 95.4%	FAP-targeted PET revealed fewer false-positive and false-negative findings than FDG PET.
Ballal et al. (35)	Median SUL_peak_ Local recurrence: 6.5 Lymph node: 6.9 Lung: 4.6 Bone: 5.8 Liver: 6.0	Primary tumour Lymph nodes Lung Pleura Bone Liver Brain	Primary tumour DR: 100% Lymph node DR: 100% Lung DR: 81.3% Bone DR: 91.6%	Lymph node DR: 98.3% Lung DR: 93.5% Bone DR: 92.4%	FAP-guided PET exhibited a superior accuracy than DOTA-NOC PET in detecting both local recurrence and distant metastases.

DR, detection rate; FAP, fibroblast activation protein; FAPi, fibroblast activation protein inhibitors; n.a., not available; PET, positron emission tomography; SUL_peak_, standardized uptake value corrected for lean body mass; SUV_max_, maximum standardized uptake value.

Only one of the included papers focused on patients diagnosed with MTC, enrolling subjects both in a staging and restaging setting. A comprehensive overview of the FAP-guided PET metrics and diagnostic accuracy reported in the paper are presented in Table 4. Concerning the diagnostic performance of the index test when compared to [^68^Ga]Ga-DOTANOC PET/CT, it showed higher accuracy in detecting both local recurrences and distant metastases (35).

With regard to FAP-guided PET imaging accuracy in different TC histopathologic subtypes, none of the included studies made a statistical analysis to assess differences in uptake values among the explored histopathologic variants. Moreover, it is worth noting that none of the included studies incorporated an immunohistochemistry (IHC) analysis in its design to assess the immunostaining of FAP in biopsy samples; in this setting, only one of the included papers performed an IHC analysis to evaluate the expression of FAP in the TME only on one pleural lesion.

Since each study explored the diagnostic yield of FAP-guided PET using a single radiopharmaceutical form, no reports are available concerning the differences in the accuracy of the different FAP-targeting radiopharmaceuticals available in this clinical setting.

## 4 Discussion

The upregulation of FAP on the cell membrane of stromal cells in the TME offers a promising opportunity for molecular imaging and potentially radioligand therapy (35). In the past few years, there has been a consistent increase in clinical research investigating the application of PET imaging using radiolabelled FAPi in several contexts, mainly in oncology. This emerging research provides crucial insights into the potential applications of this innovative diagnostic approach. Moreover, recent investigations have demonstrated that FAP-guided PET has displayed remarkable results in identifying various malignancies, including tumours frequently associated with limited or insignificant [^18^F]FDG uptake (36–38). FAP-targeted PET imaging offers several advantages, since it exhibits relatively lower levels of background activity in muscle and blood pool compared to other tracers (39).

Over the past 2 years, various clinical studies have attempted to assess the diagnostic yield of PET imaging using radiolabelled FAPi in TCs. The research aimed to determine the accuracy of FAPi in various clinical scenarios and to identify its conceivable applications for individuals diagnosed with DTC and MTC (30–35). These research papers have included newly diagnosed individuals and those who previously had surgical procedures or systemic treatments, including RAI therapy. The objective of this systematic review was to collect the existing data, analyse variations among the included studies, and advocate for future research perspectives to ultimately establish a more accurate assessment of the effectiveness of FAP-guided PET.

One of the main issues concerning the regular employment of FAP-targeting radiopharmaceuticals, both as a diagnostic probe or as a theragnostic compound, is the radiopharmaceutical retention in the target cells, especially in monomeric pharmaceutical forms, irrespectively of the radionuclide it is radiolabelled with (36). This statement opens a debate on the best uptake time to acquire emissive images. In the studies gathered in this systematic review, the uptake time ranged between 30 and 60 min, and none of them included a dynamic acquisition in their design. Theoretically, using a shorter uptake time should forestall the radiopharmaceutical excretion; however, dynamic studies of biodistribution in different neoplasms are warranted to assess the best uptake time and advocate for univocal procedure guidelines.

Concerning the diagnostic performance of FAP-targeted PET in DTC, despite most of the studies gathered in this systematic review where proof of concept trials enrolling a constrained number of DTC patients, the evidence is relatively homogeneous (30–34). Indeed, the four studies assessing the DR of this novel instrumental examination observed optimal values in neck lesions (expressed as local recurrence and lymph node metastases) as well as distant metastases in typical and atypical anatomic sites, including lung, liver, bone, and pleura. Interestingly, when compared to [^18^F]FDG PET, FAP-targeted PET imaging showed more true positive findings and fewer false negative results (30, 31, 33, 34). Despite these differences in terms of number of lesions were not statistically significant in any of the included papers, more studies are needed to assess if the increased DR of this novel imaging technique might be able to change DTC patients’ stage and, subsequently, management. The difference in terms of DR of these two instrumental examinations might underlie the variable diagnostic accuracy of [^18^F]FDG PET in DTC, which is affected by several different clinical and histological features (40–42); however, since none of the included studies assessed the differences in diagnostic accuracy of both examinations based on histologic variants and clinical features; it is not feasible to validate this hypothesis. In this setting, it is noteworthy that [^18^F]FDG has a valuable diagnostic performance in DTC patients with hematogenous metastases, as stated in a recent meta-analysis of literature (43); unfortunately, it is currently not feasible to make an indirect comparison of these two imaging techniques since the data gathered in this systematic review does not allow to pool the diagnostic accuracies reported in the included studies. More studies are needed in this setting to assess which patients might benefit from FAP-targeted imaging rather than [^18^F]FDG PET. Furthermore, given the recent technologic developments in PET devices, which allow dual-tracer acquisition protocols, future research should explore the potential impact of an imaging technique assessing two metabolic pathways (related on glucose metabolism and fibroblastic tissue remodelling, respectively) in DTC patients (44, 45).

Regarding the diagnostics of DTC, recent IHC and molecular imaging studies explored the *in vitro* and *in vivo* expression of prostate-specific membrane antigen (PSMA, also known as carboxypeptidase type II), reporting a variable expression of this transmembrane protein on the surface of neo endothelial cells within tumour’s neo angiogenesis and conflicting results concerning the potential employment of PSMA-targeted PET in DTC patients (46–48). Given the lack of existing literature comparing these two tracers in DTC patients and the recent development of bispecific radiopharmaceuticals designed to target both FAP and PSMA, it is necessary to conduct prospective studies encompassing both diagnostic methods in order to assess which instrumental examination is more dependable for this type of malignancy (49).

Only one of the included studies explored the diagnostic accuracy of FAP-targeted PET in MTC, reporting an optimal DR in the detection of neck lesions as well as distant metastases, superior to [^68^Ga]Ga-DOTA-NOC PET (35). In recent literature, it is reported that FAP is highly expressed in peritumoral and intratumoral stromal compartments of MTC and that the expression of AP positively correlates with the level of desmoplasia determined in the histological analysis (50–52). Moreover, it has been reported that desmoplasia is associated with a higher incidence of lymph node metastases in both PTC and MTC. The high uptake of radiolabelled FAPi in MTC lesions is likely explained by the stromal development and the abundant stromal components found even at the early stages of MTC onset (52). As well known, neuroendocrine neoplasms accumulate amine precursors and amino acids, including dihydroxyphenylalanine (DOPA). This molecule, labelled with [^18^F]F, has been successfully used as an imaging compound for PET/CT investigations in neuroendocrine tumours and is currently considered the best choice for imaging MTC patients in various clinical settings (53, 54). In order to assess the actual advantage of using FAP-targeting radiopharmaceuticals rather than currently available tracers in MTC and advocate for their regular employment in clinical practice, it is necessary to develop clinical trials comparing FAP-targeted imaging to [^18^F]DOPA PET in patients diagnosed with this malignancy.

So far, RAI has been the most often used additional treatment for patients with intermediate- or high-risk of disease recurrence or metastatic DTC (55). However, patients with advanced DTC either have inherent resistance or develop resistance to this treatment (56). Additionally, MTC and anaplastic TC do not exhibit a valuable response to RAI therapy due to their inability to metabolize iodine. Despite significant advancements in Tyrosine Kinase inhibitor treatments for RAI-R TC in recent years, the presence of drug resistance continues to pose a significant challenge in enhancing prognosis (57). To date, there are no alternative therapies accessible to individuals with progressive tumours who have either completed or declined traditional treatment choices (58). Based on these statements, FAP-targeted therapy with radiolabelled FAPI might provide a potential therapeutic option for RAI-R TC patients. In this setting, several [^177^Lu]Lu-radiolabelled FAP-targeting compounds were tested to assess their safety; as a result, these pharmaceuticals were well tolerated by RAI-R TC patients. Moreover, the preliminary data on the efficacy of these novel radiopharmaceuticals encourage further research to assess if they can significantly prolong survival in this clinical scenario (59–63). Table 5 synthesizes the main results of these trials.

**TABLE 5:** Synthesis of papers dealing with FAP-targeted radioligand therapy.

References	No. patients	Histopathological TC subtypes (no. patients)	Radiopharmaceutical	Main results
Ballal et al. (59)	1	1 MTC	[^177^Lu] Lu-DOTAGA.(SA.FAPi)2	Significative reduction of the tumour burden with significant improvement in the quality of life of the patient.
Ballal et al. (60)	15	15 RAI-R DTC	[^177^Lu]-DOTAGA.(SA.FAPi)2	About half of the enrolled patients had partial response to treatment or stable disease. None of the patients experienced grade III/IV hematological, renal, or hepatotoxicity.
Fu et al. (61)	1	RAI-R DTC	[^177^Lu] Lu-FAPi-46	The patient had stable disease after a four-cycles treatment.
Fu et al. (62)	12	RAI-R DTC	[^177^Lu] Lu-EB-FAPi	Overall, the radiopharmaceutical was well tolerated by all patients with high radiation doses delivered to mRAIR-TC lesions.
Martin et al. (63)	1	MTC	[^177^Lu] Lu-DOTAGA.Glu.(FAPi)_2_	The employed radiopharmaceutical showed longer retention than other pharmaceutical forms.

FAPi, fibroblast activation protein inhibitors; MTC, medullary thyroid cancer; RAI-R, radioiodine refractory; DTC, differentiated thyroid cancer.

This systematic review represents the first thorough literature examination concerning the use of FAP-targeting PET radiopharmaceuticals in patients with thyroid malignancies. However, it is worth noting that it accounts for significant limitations, including the limited number of studies in the field of interest with constrained sample sizes and their heterogeneity, which hampered the possibility of drawing up a quantitative analysis of the retrieved data. Furthermore, potential sources of bias about patient selection and comparative imaging domains were found in the included studies.

## 5 Conclusion

The presented systematic review has furnished qualitative data underscoring the potential role of FAP-targeted radiopharmaceuticals in the diagnostics of different forms of TC, and potentially in its employment as theragnostic agent.

## Data availability statement

The original contributions presented in the study are included in the article/Supplementary material, further inquiries can be directed to the corresponding author.

## Author contributions

AR: Writing – review and editing, Writing – original draft. DA: Writing – review and editing. FD: Writing – review and editing. MC: Writing – review and editing. BM: Writing – review and editing. SA: Writing – review and editing. MR: Writing – review and editing. FB: Writing – review and editing. AP: Writing – review and editing. GT: Writing – review and editing, Writing – original draft.
